# Determining the level of knowledge and consumption of probiotics and prebiotics among athletes in Jordan

**DOI:** 10.1017/jns.2025.10013

**Published:** 2025-06-16

**Authors:** Ola D. Al-Maseimi, Leena Ahmad, Nour A. Elsahoryi, Lena Al-Maaitah

**Affiliations:** 1 Department of Nutrition and Food Science, Faculty of Allied Medical Sciences, Al-Balqa Applied University, Al-Salt, Jordan; 2 Department of Nutrition, Faculty of Pharmacy & Medical Sciences, University of Petra, Amman, Jordan; 3 Department of Nutrition and Food Science, Mutah University, Al-Karak, Jordan

**Keywords:** Dietary practices, Jordanian athletes, Nutritional knowledge, Prebiotics, Probiotics, Sports nutrition, Supplement usage, PA, Physical activity, GI, Gastrointestinal, URTIs, Upper Respiratory Tract Infections, SCFAs, Short-chain fatty acids, SPSS, Statistical Package for Social Science, SD, Standard deviation, CVR, Content Validity Ratio, CVI, Content Validity Index

## Abstract

Interest in probiotics and prebiotics in sports nutrition is growing, but research on Jordanian athletes remains limited. While gut microbiota influences athletic performance, little is known about athletes’ understanding and use of probiotic- and prebiotic-rich foods in this region. This exploratory study investigates the knowledge, dietary habits, and correlations with gastrointestinal (GI) problems among Jordanian athletes to inform future research and interventions. The study provides insights into how awareness and consumption of gut-supportive foods can impact GI health, offering broader nutritional implications for global dietary strategies for athletes. A standardised questionnaire was administered to 324 athletes (ages 18–22) to assess knowledge, dietary practices, and GI symptoms. Descriptive statistics summarised the data, and chi-square tests examined associations among knowledge, diet, and GI symptoms (p < 0.05). Knowledge and diet were scored based on correct responses and reported intake of gut-supporting foods. Results showed that 55.9% of athletes were familiar with probiotics and 32.1% with prebiotics. The majority reported low consumption of probiotic- and prebiotic-rich foods, with 72.2% scoring low on diet intake. Although 60.5% seldom consumed fast food, overall intake of gut-supportive foods was limited. No statistically significant associations were found, but a weak positive trend between dietary habits and GI symptoms was observed, suggesting diet may have a modest influence on GI health. Living arrangements influenced both dietary choices and GI symptoms. This study highlights the need for targeted nutrition education to improve awareness and intake of probiotics and prebiotics, potentially supporting GI health and enhancing athletic performance.

## Introduction

Physical activity (PA), which encompasses any sort of movement in which the contraction of skeletal muscles increases energy consumption, is recognised to provide several health advantages.^(1)^ The main goal of physical exercise is to increase physical functionality through adaptation. Furthermore, physical exercise appears to boost microbial diversity in the gut, increase the Bacteroidetes:Firmicutes ratio, encourage the development of bacteria that can influence mucosal immunity, and improve intestinal barrier functions — all of which are advantageous to the host’s health.^(2)^

Athletes have very specific needs for nutrition to help performance, recovery, and health in general. Probiotics and prebiotics as dietary factors have been studied extensively for their contribution to gut health, immunity, and metabolic function.^(3,4)^ Research in Western populations has shown probiotic use to decrease exercise-induced gastrointestinal (GI) symptoms, increase nutrient absorption, and improve immune function.^(5,6)^ GI disturbances are also common in this group due to intensive training, dehydration, and diet composition that detrimentally affect performance.^(7)^

Nevertheless, there is a clear gap in current literature regarding the knowledge, consumption patterns, and perceived benefits of probiotics and prebiotics among Middle-Eastern athletes, including those in Jordan. Most existing studies focus on Western dietary patterns, where probiotic-rich foods are more commonly consumed.^(8)^ In contrast, Jordanian athletes may have limited awareness and access to such foods due to different dietary habits, food environments, and cultural practices.

Understanding athletes’ knowledge and consumption of probiotics and prebiotics in Jordan may support the development of targeted nutritional interventions tailored to their specific needs.

Highly aerobic endurance athletes are at a higher risk of leaky gut and intestinal permeability because of exercise-induced stress, which reduces GI blood flow, causing inflammation and gut wall damage.^(5,9)^

Short-chain fatty acids (SCFAs) produced by beneficial bacteria significantly contribute to the health of the intestinal wall and play a role as a barrier, preventing the passage of harmful substances.^(10)^ According to supporting evidence, an increase in dietary fibre is also related to increased microbial diversity and richness in individuals who lack diversity of bacteria in their gut.^(8)^ This research highlights how crucial it is for athletes to consume prebiotic and probiotic foods to keep a diverse and balanced gut microbiome.

In recent years, researchers have focused on the health of athletes to prevent or manage exercise-related health disorders through the use of dietary supplements.^(11)^ Additionally, studies have demonstrated that probiotics can decrease the likelihood of GI and respiratory infections as well as the severity of symptoms related to these conditions in athletes.^(12)^ As a result, probiotic supplementation may benefit athletes’ health.^(6)^

Although several bacteria are utilised as probiotics, the most common strains are *Lactobacillus*, *Enterococcus*, *Bifidobacterium*, *Propionibacterium*, and *yeasts* such as *Saccharomyces boulardii*.^(13)^ *Lactobacillus* and *Bifidobacterium* strains show potential benefits for athletes and fitness enthusiasts by improving nutrient absorption, energy metabolism, and exercise performance while reducing oxidative stress and inflammation.^(14)^

Probiotic supplementation also boosts amino acid absorption from plant proteins and enhances glucose absorption and oxidation during exercise.^(3,15)^ Microbes in the intestinal microbiota perform metabolic processes such as converting carbohydrates into SCFAs, lipid metabolism, and vitamin synthesis, promoting immune system maturation, and protecting against pathogens.^(16)^

Limited knowledge of probiotics and prebiotics among athletes may hinder their ability to optimise gut health, which is important for digestion, immune function, and nutrient absorption, all of which are critical to training adaptation and recovery.^(6,11)^ In addition, probiotics and prebiotics are known to enhance immune function, reduce inflammation, and improve endurance performance; thus, they are important for an athlete’s diet.^(5,17)^ This lack of awareness in this area indicates the need for specific educational interventions aimed at optimising gut health and performance in athletic populations.

Thus, the objective of this study is to evaluate the knowledge and consumption of probiotics and prebiotics among Jordanian athletes and to determine if a greater knowledge level is associated with higher dietary intake and fewer GI symptoms. It also examines the potential associations among knowledge, diet quality, and GI symptoms. The findings will contribute to developing targeted nutrition education programmes to enhance athletes’ gut health and overall well-being.

## Methodology

### Participant selection

The study was carried out in Jordan, using athletes as the target population. The study included athletes in Jordan who met the following inclusion criteria: (1) 18–22 years old, (2) trained physically in a structured manner at least three times a week, and (3) participated in recreational or competitive organised sports. This definition is consistent with previous literature that considers athletes as individuals who train regularly to improve performance.^(18)^ A screening question in the questionnaire compelled participants to acknowledge that they were eligible before continuing. Those who answered ‘Yes’ were the only ones permitted to move forward and take the survey to keep the study specific to the desired athlete population.

The 18–22 age bracket had been chosen to assess a relatively homogenous sample as it is known that athletes below 18 years old present different metabolic and nutritional requirements due to ongoing growth and development, whilst over 22 years old athletes will tend to undertake more independent feeding habits and training commitments will be more diverse.^(19)^ This age range was selected to increase the homogeneity of the participants’ knowledge on probiotics and prebiotics within an organised sports setting. The survey was shared through Facebook, Instagram, and WhatsApp. This approach helped capture a broad and diverse group of athletes.

We used a cross-sectional approach to investigate particular parameters of interest in this demographic. To achieve adequate representation, the study used an online questionnaire that was disseminated using popular social media platforms like Facebook, Instagram, and WhatsApp. The high internet penetration rate in Jordan exceeds 90% of the population, and social media is used extensively among young adults, with university students and athletes being the most active users.^(20)^ As social media is one of the primary tools of communication used by this demographic, its usage allowed for broad participation across sports. Moreover, the total number of samples collected (N=324) was more than N (279), which confirms the efficiency of the method of data collection used.

This study adhered to the ethical principles outlined in the Declaration of Helsinki and received approval from the Ethical Committee of the University of Petra, Jordan (approval number: S/6/10/2023).

The study questionnaire was anonymous and voluntary, with no requests for personal information. The participants were notified in writing about the anonymous collection and particular use of the data for the study’s purposes. Informed electronic consent was obtained from all participants before completing the questionnaire. Respondents had to confirm their consent to continue. As no personally identifiable or sensitive data were collected, the research ethics committee did not require a separate signed consent document.

### Sample size

To determine the sample size, we assumed a 50% prevalence of the factor of interest among the athletic population. Following the previously described approach, the required sample size was determined to be 279, taking into account a 90% confidence interval, an expected proportion of 0.5, and a margin of error (precision) of 0.5.^(21)^ This method created a solid foundation for statistical analysis, allowing us to safely state the presence of the factor in the research population with a 90% confidence level, as indicated by a confidence range spanning from 45% to 55%.

### Data collection

The data collection involved distributing and sharing an online questionnaire across major social media channels during a 3-month period from the 21st of October 2023 to the 21st of January 2024 to ensure a varied and representative sample. In all, 324 responses were obtained from the athletes who actively participated in the study. Importantly, the actual sample size exceeded the needed sample size, demonstrating the reliability of our data collection method. Furthermore, participants in this study did not need to provide consent.

### The study questionnaire

The questionnaire was developed in English following previous methods^(9)^ and translated into Arabic to facilitate effective communication between the data collectors and the respondents. This marked the inaugural translation of such a questionnaire into Arabic for use in Jordan, an Arabic-speaking nation.

The current study translated the questionnaire into Arabic using the forward-backward technique, which was recommended in a previous research.^(22)^ The survey was originally translated from English to Arabic by a bilingual researcher who was fluent in both languages. After that, a second researcher who was multilingual translated the questionnaire backwards from Arabic to English. The original and back-translated English translations were then carefully reviewed to determine whether the item semantics had been accurately preserved.

The questionnaire included ten questions designed to assess athletes’ prior clinical history, types and frequencies of GI disturbances, dietary preferences, and supplementation practices. These questions aimed to capture relevant health and diet-related factors affecting athletes. The questionnaire was structured to ensure comprehensive data collection on these aspects. A pilot sample comprising twenty individuals was recruited to assess the Arabic version of the questionnaire to validate its psychometric qualities. The pilot research was carried out from 7 October 2023 to 20 October 2023. Ten working days after the initial tool submission, participants were recontacted and sent the questionnaire once again via the WhatsApp messaging application. Reliability was assessed through internal consistency (Cronbach’s alpha) for the knowledge (questions 7 and 10) and diet (questions 18 and 19) sections based on the original questionnaire scores. The content validity was calculated from the critical values for Lawshe’s content validity ratio^(23)^. The questionnaire needs approximately 10 min, and to safeguard participant anonymity, unique identifier codes were assigned to the questionnaires.

### Assessment of knowledge and diet categories

Participants’ responses to specific items in the questionnaire were used to assess knowledge and diet categories. The knowledge scores were tracked based on responses to questions that assessed awareness of probiotics and prebiotics, including definitions, sources, and associated health benefits. Correct answers were scored with a total of 18 points. Participants were classified into the following categories: low knowledge (0–6 correct answers), moderate knowledge (7–12 correct answers), and high knowledge (13–18 correct answers).

Diet categories were directly assessed according to the consumption of probiotic- and prebiotic-containing foods in responses (questions 18 and 19). Participants chose foods that they consumed regularly, resulting in a maximum score of 12 points. It was classified as a low diet score (0–4 selected food items), a moderate diet score (5–8 selected food items), and a high diet score (9–12 selected food items).

These categorizations allowed for statistical analysis to determine associations between knowledge, dietary intake, and GI symptoms, using chi-square tests to evaluate significance at p < 0.05.^(24,25)^

### Additional variables considered

Living arrangements were included as an additional variable, as they may affect dietary choices, meal regularity, and stress levels, all of which can influence GI symptoms and gut health.^(5)^ Therefore, the relationship between living arrangements, GI symptoms, and intake of probiotic and prebiotic foods was analysed.

Additionally, dietary supplement usage was considered. The use of probiotics, prebiotics, and other performance-related supplements (e.g. multivitamins, protein powders, pre-workout formulas) was recorded to identify trends in gut health management and nutritional habits. Inclusion of these variables aimed to provide a more comprehensive overview of factors affecting athletes’ dietary behaviour and GI well-being beyond knowledge alone.

### Statistical method

The data editing process involved thorough examination and validation of the completed questionnaires both at the conclusion of individual interviews and at the end of the entire survey, which were conducted prior to the subsequent analysis. Descriptive statistics were used to present the data, including frequencies (N) and percentages (%) for categorical variables, as well as means and standard deviations for continuous variables. Chi-square tests were performed to examine whether significant associations existed between levels of knowledge, dietary intake, and reported GI symptoms. The reliability of the questionnaire was assessed using Cronbach’s alpha, and validity was evaluated using the content validity ratio (CVR) and content validity index (CVI). Comparisons of probiotic and prebiotic food consumption across different living arrangements were analysed using cross-tabulations and percentage distributions to identify dietary patterns and variations. All analyses were conducted using SPSS version 25.0, with statistical significance set at p < 0.05.

## Results

### Reliability and psychometric validation of the study questionnaire

The questionnaire exhibited high reliability in terms of overall internal consistency, with a Cronbach’s alpha coefficient of 0.72 for the knowledge score and 0.76 for the diet score, indicating that the items are sufficiently consistent to indicate that the measure is reliable. To ensure the accuracy of the Arabic questionnaire, a panel consisting of ten food science lecturers was used to validate the questionnaire. In the content validity assessment, a panel of ten experts (Expert 1 to Expert 10) evaluated the various questionnaire items. All the experts, except for Expert 9, marked Item 1 as relevant, resulting in a CVR of 1. All the experts, including Expert 10, indicated the relevance of Item 2, yielding a CVR of 1. There was some variability in the opinions of the experts regarding the incorporation of specific foods into the diet. Experts 1 through 8 and Expert 10 marked the item as relevant, resulting in a CVR of 0.636. The experts’ evaluations for Item 4 were more consistent with all the experts, except for Expert 9, who indicated that the item was relevant. This led to a CVR of 0.818. The critical value for relevance for a panel size (N) of 11 is 0.636. The CVI for the overall assessment is 0.864, indicating a strong level of content validity. The results suggest a high degree of agreement among the experts regarding the relevance of most questionnaire items, as evidenced by the CVRs and the overall CVI.

### Demographic characteristics

Tables 1–3 represent the sample count and percentage of the population for selection per questionnaire answer: demographic information, knowledge, and health and diet. Table 1 displays the responses to questions 1–3, emphasising demographic details. The age range of the individuals I included in the survey was 18–22 years, with an average age of 20.83 ± 1.20 years. Among the 324 participants, a significant portion engaged predominantly in cardio exercises (30.2%) and football (11.1%).

**Table 1.** Demographic information (N =324)

Characteristics	Categories	Frequency	Percent (%)
Age (years)	18.00	16	4.9
	19.00	38	11.7
	20.00	54	16.7
	21.00	94	29.0
	22.00	122	37.7
Gender	Female	228	70.4
	Male	96	29.6
Education	Secondary school or less	25	7.7
	Undergraduate	280	86.4
	Postgraduate	19	5.9
Sport	I prefer not to answer	58	17.9
	Cardio exercises	98	30.2
	Football	36	11.1
	Track and field	63	19.4
	Swimming	48	14.8
	Tennis	5	1.5
	Shooting sports	13	4.0
Living arrangements	Off-campus family home	290	89.5
	Off-campus apartment	24	7.4
	On campus with dining plan	10	3.1

**Table 2.** Knowledge questions with correct response indication (N = 324)

Question	Response option	Correct?	Count	Percent (%)
Q4: What is the daily recommended intake for fibre in your diet?	3–5 g	❌	88	27.2
	10–15 g	❌	23	7.1
	25–30 g	**✅**	188	58.0
	50–70 g	❌	25	7.7
Q5: True or false. It is bad to have a wide variety of bacteria living in your gut.	True	❌	235	72.5
	False	**✅**	89	27.5
Q6: Which of the following describes the term ‘probiotic’?	Live organisms that benefit your health	**✅**	181	55.9
	Substance used by organisms	❌	96	29.6
	Biological macromolecule	❌	47	14.5
QQ 7: Select all of the following sources of probiotic foods:^a^	Greek yogurt	**✅**	183	56.5
	Kombucha	**✅**	41	12.7
	Cottage cheese	**✅**	203	62.7
	Banana	❌	118	36.4
	Regular yogurt	**✅**	273	84.3
	Dark chocolate	**✅**	141	43.5
	Cheddar cheese	**✅** (debatable)	78	24.1
	Kefir	**✅**	65	20.1
	Jerusalem artichoke	❌	28	8.6
Q8: Which of the following are true of probiotics? Select all that apply^a^	Improves immune response	**✅**	185	57.1
	Antimicrobial activity	**✅**	158	48.8
	Reduces cholesterol	**✅**	62	19.1
	Prevents antibiotic diarrhoea	**✅**	143	44.1
	10^8^–10^9^ every 6 weeks to show effect	**✅**	64	19.8
	All supplements FDA approved	❌	97	29.9
Q9: Which of the following describes the term ‘prebiotic’?	Live organisms	❌	166	51.2
	Substance used by organisms	**✅**	104	32.1
	Biological macromolecule	❌	54	16.7
Q10: Select all of the following sources of prebiotic foods:^a^	Soybeans	**✅**	114	35.0
	Oats	**✅**	128	39.3
	Wheat	**✅**	97	29.8
	Legumes	**✅**	93	28.5
	Asparagus	**✅**	49	15.0
	Pickles	❌	126	38.7
	Sauerkraut	❌	120	36.8
	Onions	**✅**	89	27.3
	Dark chocolate	**✅**	64	19.6
Q11: Which of the following are the health benefits of prebiotics? Select all^a^	Reduces inflammation	**✅**	138	42.6
	Improves the immune system	**✅**	244	75.3
	Increases calcium absorption	**✅**	87	26.9
	Decrease in allergies	**✅**	79	24.4
	Slows metabolism	❌	40	12.3
	Always aids weight loss	❌	95	29.3
Q12: True or false. Higher levels of physical activity correspond to greater microbial diversity in the gut.	True	**✅**	101	31.2
	False	❌	223	68.8

a Multiple response.

**Table 3.** Health and diet questions (N =324)

Question/response	Count	Percent (%)
Q13: Have you ever previously been clinically diagnosed with any of the following digestion-related conditions?^a^		
No	199	61.4
Irritable bowel syndrome	59	18.2
Dairy intolerance	24	7.4
Gluten intolerance	21	6.5
Yes, but not listed	17	5.2
Chron’s disease	13	4.0
Celiac disease	23	7.1
I prefer not to answer	25	7.7
Q14: Do you currently experience any of the following symptoms on a regular basis?^a^		
I do not experience any of these symptoms	762	85.2
Bloating	45	13.9
Gas	38	11.7
Upset stomach	37	11.4
Diarrhoea	46	14.2
Constipation	36	11.1
Heartburn	35	10.8
I prefer not to answer	0	0
Q15: If you answered yes in the previous question, how often do you experience the selected symptom?		
I did not answer yes	146	45.1
Everyday	35	10.8
2–3 times per week	75	23.1
4–5 times per week	22	6.8
5+ timers per week	12	3.7
I prefer not to answer	34	10.5
Q16: Have any of the previous selected symptoms prevented participation in a game, match, or training session?		
Yes, but rarely	65	20.1
Yes, frequently	15	4.6
Never	206	63.6
I prefer not to answer	38	11.7
Q17: How often do you eat fast food or take out per week?		
Rarely or less than once per week	196	60.5
2–3 times per day	4	1.2
2–3 times per week	83	25.6
4–5 times per week	30	9.3
I prefer not to answer	11	3.4
Q18: Which of the following foods do you regularly incorporate into your diet?^a^		
Bananas	115	35.5
Whole oats	76	23.5
Onions	149	46.0
Garlic	113	34.9
Soy	20	6.2
Jerusalem artichoke	24	7.4
I prefer not to answer	0	0
Q19: Which of the following foods do you regularly incorporate into your diet?^a^		
Yogurt	199	61.4
Cheddar cheese	52	16.0
Pickles	113	34.9
Kombucha	9	2.8
Cottage cheese	145	44.8
Sauerkraut	28	8.6
I prefer not to answer	0	0
Q20: Do you currently take a probiotic supplement?		
Yes	60	18.5
No	264	81.5
Q21: Do you currently take a fibre or prebiotic supplement?		
Yes	50	15.4
No	274	84.6
Q22: Do you currently take a multivitamin, pre-workout, protein, creatine, or any other supplements?		
Yes	105	32.4
No	219	67.6

a Multiple response.

### Knowledge of probiotics and prebiotics

This survey included nine questions specifically designed to assess participants’ knowledge of probiotics and prebiotics, focusing on their definitions, sources, and associated health benefits. The findings, outlined in Table 2, reveal uneven levels of understanding among the athletes. While 58.0% selected ‘25–30’ (the stated answer for fibre intake) as the daily recommendation, only 27.5% correctly recognised the significance of gut bacterial diversity. Knowledge regarding food sources was similarly variable: 84.3% identified regular yogurt as a probiotic source, yet only 8.6% recognised Jerusalem artichoke. In addition, dark chocolate was selected by 19.6% of respondents, whereas soybeans and pickles were chosen by 35.0% and 38.7%, respectively. Furthermore, when asked about the relationship between PA and microbial diversity, 68.8% erroneously responded that there was no association, with only 31.2% affirming the correct relationship.

### Health and dietary patterns among athletes

In a comprehensive exploration of health and dietary habits among a sample of athletes (N = 324), Table 3 illuminates insights emerged from ten thoughtfully designed questions. The survey focused on prior clinical history, types and frequencies of GI disturbances, dietary preferences, and practices related to supplementation, presenting a vibrant mosaic of data. Regarding the history of digestive conditions (Q13), the majority (61.4%) reported no prior clinical diagnosis, with noteworthy cases including irritable bowel syndrome (18.2%), dairy intolerance (7.4%), and gluten intolerance (6.5%). The significant findings included 40.7% reporting no regular symptoms, while others highlighted bloating (24.7%), gas (18.8%), and upset stomach (24.7%) through Q14. The frequency of symptoms (Q15) ranged widely, from every day (10.8%) to 5+ times per week (3.7%), revealing the spectrum of experiences. A prevailing trend of consuming fast food rarely or less than once per week (60.5%) was observed. The answers to the supplemental practice questions (Q20, Q21, and Q22) indicated that 18.5% of the probiotic supplements were taken, while 81.5% were not taken. Fibre or prebiotic supplements were embraced by 15.4%, with 84.6% opting against them. In the broader supplement landscape, 32.4% of the participants used multivitamins, pre-workouts, protein, creatine, or other supplements, while 67.6% did not.

### Knowledge and diet scores

Table 4 shows the distribution of participants across knowledge and diet categories. In terms of knowledge, 57.4% of the participants fell into the low category, 42% demonstrated a moderate level, and a substantial 0.6% exhibited a high level. In the realm of diet, 72.2% indicated a low score, 25.9% a moderate score, and an impressive 1.9% a high score

**Table 4.** Percentage of the knowledge and diet categories^a^ N (%)

	Low %	Moderate%	High%	Score mean±SD
Knowledge	186 (57.4%)	136 (42%)	2 (0.6%)	6.17±2.20
Diet	234 (72.2%)	84 (25.9%)	6 (1.9%)	3.22±2.18

a *Note*: Knowledge categories were defined as follows: low = 0–6 correct answers, moderate = 7–12, high = 13–18. Diet categories were defined as follows: low = 0–4 prebiotic/probiotic foods consumed, moderate = 5–8, high = 9–12.

### Cross-comparison between knowledge, diet, and GI Symptoms

Cross-comparison between knowledge, diet, and GI symptoms was explored to determine potential associations and help evaluate the initial hypotheses.

The association between knowledge and diet categories was first examined to determine whether knowledge about prebiotics and probiotics significantly influenced dietary choices. Table 5 presents the relationship between knowledge and diet categories as determined by the chi-square test. The distribution of participants in the low knowledge category was as follows: 131 participants reported a low diet, 102 participants a moderate diet, and 1 participant a high diet. For those with moderate knowledge, 53 participants had a low diet, 30 had a moderate diet, and 1 had a high diet. In the high knowledge category, 2 participants reported a low diet, 4 participants a moderate diet, and none reported a high diet. In total, there were 186 participants in the low knowledge category, 136 in the moderate knowledge category, and 2 in the high knowledge category, for a total of 324 participants.

**Table 5.** Chi-square of the knowledge versus diet

		Knowledge categories			
		Low	Moderate	High	Total
Diet categories	Low	131	102	1	234
	Moderate	53	30	1	84
	High	2	4	0	6
Total		186	136	2	324

Chi-square = 3.58, p-value = 0.47.

The association between GI symptoms and diet categories was also analysed to assess whether dietary intake of prebiotic and probiotic foods correlated with the presence of GI issues. Table 6 illustrates the association between GI symptoms and different diet categories, as examined through the chi-square test. In the low diet category, out of 234 participants, 193 reported no GI symptoms, and 41 reported experiencing such symptoms. Among those in the moderate diet category, 84 participants were in total, 78 reported no GI symptoms, and 6 reported experiencing them. In the high diet category, which consisted of 6 participants, 5 reported no GI symptoms, and 1 reported experiencing them. The overall counts across all diet categories showed that out of 324 participants, 276 reported no GI symptoms, and 48 reported experiencing such symptoms. The chi-square analysis yields a value of 5.29 with a corresponding p-value of 0.07. While the association between GI symptoms and diet categories is not as pronounced as that observed with knowledge levels in Table 7. A p-value of 0.07 indicates that this association is not statistically significant at the conventional significance level of 0.05.

**Table 6.** Chi-square of the GI symptoms versus diet

		GI Symptoms		
		No	Yes	Total
Diet categories	Low	193	41	324
	Moderate	78	6	84
	High	5	1	6
Total		276	48	324

Chi-square = 5.29, p-value = 0.07.

**Table 7.** Chi-square of the GI symptoms versus knowledge

		GI symptoms		
		No	Yes	Total
Knowledge categories	Low	160	26	186
	Moderate	115	21	136
	High	1	1	2
Total		276	48	324

Chi-square = 2.11, p-value = 0.35.

The association between GI symptoms and knowledge levels was examined to determine whether awareness of probiotics and prebiotics correlates with digestive health status. Table 7 presents the analysis of the association between GI symptoms and knowledge levels using the chi-square test. In the low knowledge category, consisting of 186 participants, 160 reported no GI symptoms, while 26 reported experiencing such symptoms. Among those with moderate knowledge (136 participants in total), 115 reported no GI symptoms, and 21 reported experiencing them. In the high knowledge category, which included 2 participants, 1 participant reported no GI symptoms, and 1 reported experiencing them. Across all knowledge categories, out of 324 participants, 276 reported no GI symptoms, and 48 reported experiencing such symptoms.

The chi-square test yields a value of 2.11 with a corresponding p-value of 0.35. The results indicate that there is no statistically significant association between GI symptoms and knowledge levels at a conventional significance level of 0.05.

### Supplement usage among athletes

**Fig. 1.**
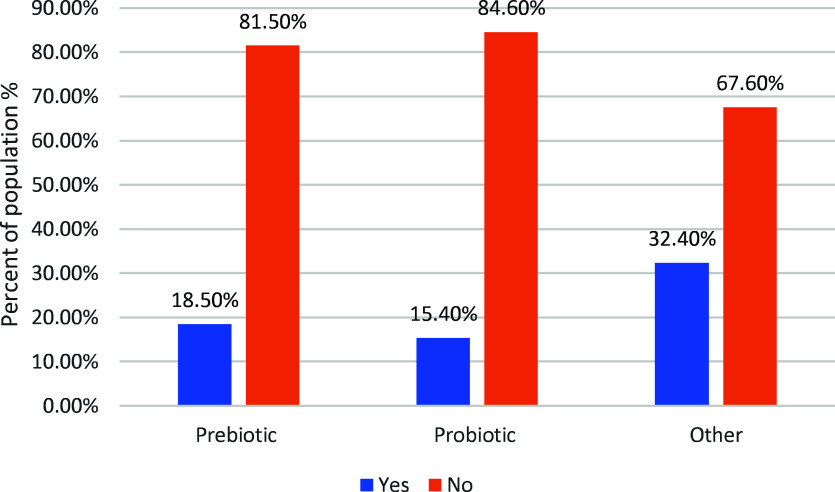
Supplement usage among study participants.

Figure 1 indicates that incorporating prebiotic supplements was observed among 18.50% of participants, with the majority (81.50%) opting not to include them. Regarding probiotic supplements, 15.40% of participants integrated them into their routine, while 84.60% did not partake in probiotic supplementation. In the realm of other supplements (such as multivitamins, pre-workouts, proteins, creatine, etc.), 32.40% of participants included them in their regimen, whereas 67.60% did not use additional supplements regularly.

### Impact of living arrangements on GI symptoms, probiotic, and prebiotic intake

**Fig. 2.**
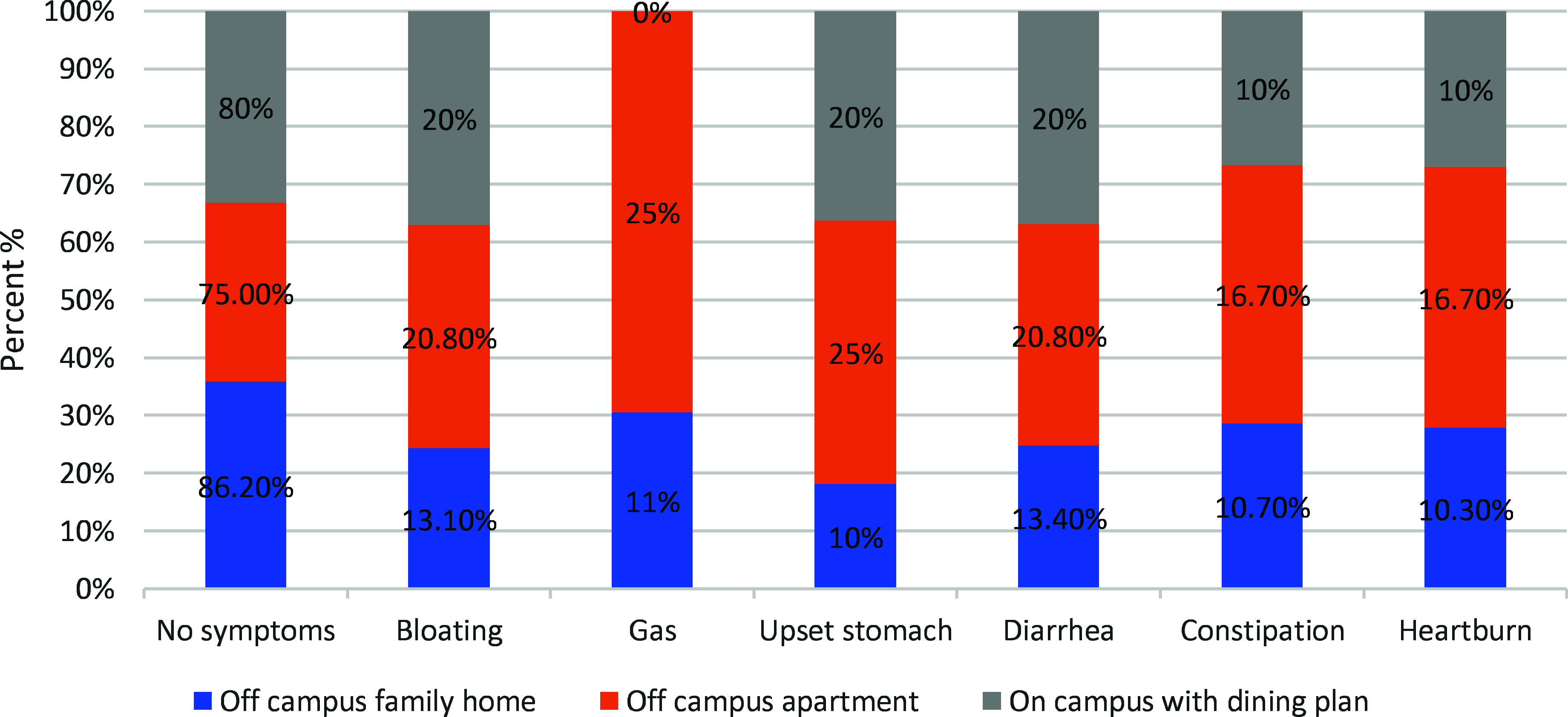
Prevalence of gastrointestinal (GI) symptoms by living arrangement.

The impact of living arrangements on GI symptoms, probiotic, and prebiotic intake was evident across the study sample. Those who did not experience any of the listed symptoms constituted 14.8% of the total respondents (48 individuals). On the other hand, individuals reporting symptoms made up 85.2% of the total (276 individuals). Figure 2 displays the prevalence of GI symptoms across different living arrangements. Among the off-campus family homes, 86.20% of the individuals reported no symptoms, 13.10% experienced bloating, 11.00% reported gas, 10.00% had an upset stomach, 13.40% suffered from diarrhoea, 10.70% faced constipation, and 10.30% had heartburn. For the off-campus apartments, 75.00% of the patients had no symptoms, 20.80% had bloating, 25.00% had gas, 25.00% had an upset stomach, 20.80% had diarrhoea, 16.70% had constipation, and 16.70% had heartburn. According to the on-campus housing with a dining plan, 80.00% of the participants reported no symptoms, 20.00% experienced bloating, 0.00% reported gas, 20.00% had an upset stomach, 20.00% faced diarrhoea, 10.00% suffered from constipation, and 10.00% had heartburn. These figures highlight the variability in the prevalence of GI symptoms among individuals living in different arrangements.

**Fig. 3.**
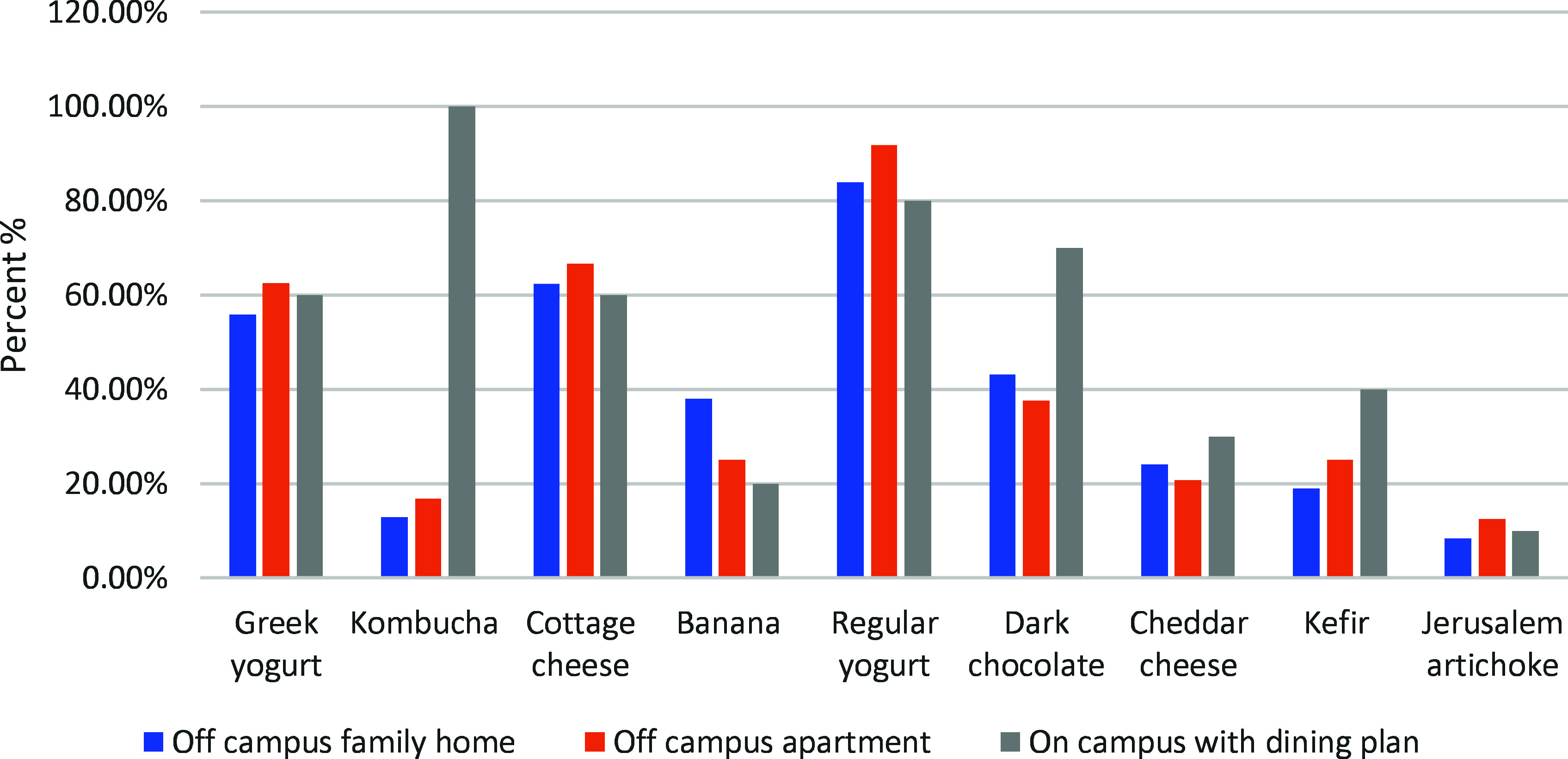
Probiotic food consumption patterns across living arrangements.

Probiotic intake and living arrangements also demonstrated notable differences. The distribution of probiotic food consumption according to each living arrangement is shown in Fig. 3. The prevalence of Greek yogurt, kombucha, cottage cheese, regular yogurt, dark chocolate, cheddar cheese, and kefir use among off-campus family home participants were 55.90%, 12.80%, 62.40%, 83.80% 43.10%, 24.10%, 19% respectively.

According to off-campus apartment data, 62.50% of respondents reported consuming Greek yogurt, 16.70% reported consuming kombucha, 66.70% reported consuming cottage cheese, 91.70% reported consuming regular yogurt, 37.50% reported consuming dark chocolate, 20.80% reported consuming cheddar cheese, and 25% reported consuming kefir.

Of participants in on-campus housing with a dining plan: 60% Greek yogurt, 100% kombucha, 60% cottage cheese, 80% regular yogurt, 70% dark chocolate, 30% cheddar cheese, 40% kefir.

**Fig. 4.**
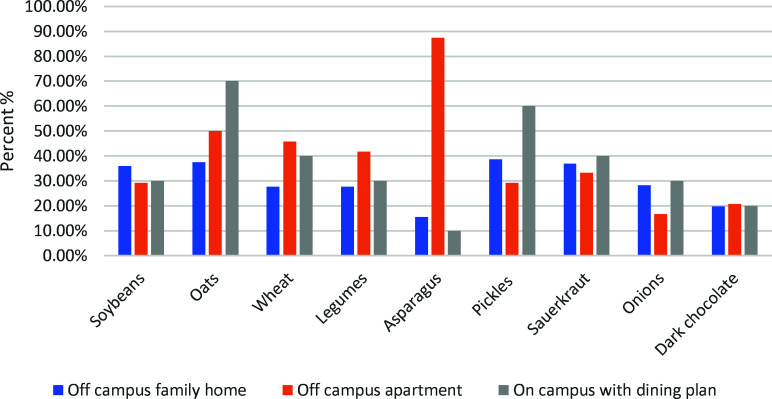
Prebiotic food intake by residential living arrangement.

Prebiotic intake and living arrangements followed a similar trend. Figure 4 illustrates the correlation between the place of residence and the inclusion of prebiotic foods in the diet. The percentages represent the prevalence of various prebiotic-rich items among individuals in different living arrangements. In off-campus family homes, 35.90% had soya, 37.60% consumed oats, 27.60% had wheat, 27.60% had legumes, 15.50% had asparagus, 38.60% had pickles, 36.90% had sauerkraut, 28.30% had onions, and 19.70% had dark chocolate.

Among off-campus apartments individuals, soybeans, oats, wheat, legumes, asparagus, pickles,  sauerkraut, onions, and dark chocolate intake were 29.20%, 50%, 45.80%, 41.70%, 87.50%, 29.20%, 33.30%, 16.70%, and 20.80%.

Of those who were on-campus housing with a dining plan, 30% ate soybeans, 70% ate oats, 40% ate wheat, 30% ate legumes, 10% ate asparagus, 60% ate pickles, 40% ate sauerkraut, 30% ate onion, and 20% ate dark chocolate.

## Discussion

This study aimed to assess the level of knowledge, dietary habits, and GI health among Jordanian athletes about probiotics and prebiotics. The findings revealed a clear knowledge gap, as many athletes were unfamiliar with key terms and concepts. No significant associations were observed between knowledge levels and dietary choices or between knowledge and GI symptoms. However, a potential trend suggested that dietary habits may influence the occurrence of GI symptoms, warranting further investigation. Although GI symptoms were frequently reported, most athletes did not perceive these symptoms as affecting their athletic performance.

Understanding of probiotics and prebiotics among athletes remains an area of global concern, reflecting gaps in nutritional education and awareness. Our results align with previously published studies indicating limited knowledge of probiotics and prebiotics among athletes globally. For instance, a survey found that 85% of participants reported knowing very little or nothing about probiotics.^(26)^.  In the United States, one report indicated that college athletes lacked even basic nutritional education, which led to poor dietary choices.^(27)^ In Europe, awareness of probiotics was reported to be between 60% and 70%, but actual incorporation into diets remained low.^(3)^

Such a difference stresses the importance of educational strategies to improve athletes’ knowledge regarding probiotics and prebiotics. While the research is in its infancy, a disconnect between the recent advances of the gut biome and practical implementation will stifle the adoption of dietary approaches that support gut health, immune response, and overall performance.^(6)^

GI health is a prevalent concern among athletes and can significantly impact both training and performance. Previous studies have reported that a notable proportion of athletes experience GI disturbances during training or competition.^(24)^

Previous research has shown that 30–70% of athletes — particularly those involved in endurance sports and weightlifting — experience some form of GI disruption.^(28)^ The current results affirm the high prevalence of GI symptoms among Jordanian athletes and suggest that these symptoms are not solely dependent on sport type. Instead, they likely reflect a combination of physiological and lifestyle factors. This aligns with prior literature indicating that GI disturbances in athletes are multifactorial in nature, influenced by training intensity, psychological stress, hydration habits, and dietary patterns^(29–31)^. As dietary and gut health continue to gain attention in the context of athletic performance, a more coordinated and individualised approach to managing GI symptoms is warranted. Therefore, sports nutritionists are encouraged to incorporate tailored interventions that support digestive health, such as the inclusion of probiotic and prebiotic foods, hydration strategies, and scheduled meal timing. Further research is recommended to assess the effectiveness of these personalised nutritional approaches in reducing GI symptoms and enhancing performance outcomes among athletes.

This study aimed to explore whether GI symptoms in Jordanian athletes are influenced by their knowledge and intake of prebiotic- and probiotic-rich foods. While no significant association was found, the findings underscore the importance of targeted nutritional education and intervention strategies to enhance athletes’ understanding of gut-friendly foods and improve dietary patterns, which in turn could support better digestive health and athletic performance.

Cross-comparison between knowledge, diet, and GI symptoms reveals important behavioural insights. In this sample, knowledge of probiotics and prebiotics did not appear to directly influence athletes’ dietary habits or digestive health. However, a possible link was observed between dietary patterns and GI symptoms, suggesting that diet may play a role in managing digestive issues, even when knowledge is present.

The absence of a clear relationship between knowledge and behaviour suggests that other factors may influence food choices more strongly — such as access to probiotic-rich foods, cultural eating habits, or affordability. Previous research has shown that nutritional knowledge alone often fails to change behaviour unless accompanied by environmental support or structured intervention programmes.^(24,25)^

These findings align with previous studies reporting limited awareness of prebiotic and probiotic sources and benefits among athletes, and they confirm that GI issues remain common within this population. Despite moderate to high knowledge levels and good dietary adherence in many participants, improvements in digestive health were not clearly observed. This suggests that knowledge alone may not lead to meaningful behaviour change or symptom relief.

Future research should investigate other potential contributing factors that may play a more influential role in the prevalence of GI symptoms among athletes, such as nutrient timing — whether athletes take probiotics or prebiotics may play a role in digestion; training intensity and stress, as high-stress training environments may exacerbate GI issues; and the gut microbiome, where analysis could discover if certain bacterial strains enhance digestion in athletes.

Furthermore, targeted interventions of nutrition education should be conducted in order to establish if increasing the probiotic/prebiotic awareness can contribute to quantifiable changes in dietary practices through the years. A comprehensive assessment may also offer a more accurate representation of the amount of understanding of prebiotic and probiotic foods.

Supplement usage among athletes appears to prioritize performance over digestive health. In this study, athletes showed a greater tendency to use general supplements, which aligns with previous studies reporting that athletes generally give more preference to supplements that directly enhance muscle recovery, energy metabolism, and performance compared to those that specifically support gut health.^(5,11)^ This pattern may reflect limited awareness among athletes of the role of gut microbiota in exercise adaptation, immune function, and nutrient absorption, which could explain the preference for performance-oriented supplements over those targeting digestive health.^(6,32)^

Living arrangements may influence dietary habits and digestive health among athletes. Variability in GI symptoms and the intake of probiotic and prebiotic foods appears to be linked to living conditions. These differences in symptoms and dietary practices may be influenced by several contextual factors, including access to food, availability of cooking facilities, and stress associated with independent living or residence hall environments. Studies have shown that athletes living in stable home environments are more likely to eat healthy meals than those in independent living conditions, which may affect gut health and dietary behaviours.^(4)^ Furthermore, the relationship between living arrangements and dietary habits may be altered by socioeconomic status, cooking skills, and access to fermented, probiotic-rich, fresh foods, which warrants further investigation in future studies. This trend needs further assessment with a greater number of participants for a more robust outcome.^(4,33)^

GI symptoms across different living arrangements were found to be more common among athletes who live off-campus or by themselves, showing an increase in GI symptoms, likely a result of spontaneous meal patterns, heavy consumption of processed foods, and heightened stress levels. These issues may be further exacerbated by limited fibre intake and suboptimal food safety practices.^(5)^ In contrast, those who live with family enjoy structured meals abundant in gut-supporting nutrients with lower GI discomfort.

Probiotic intake and living arrangements showed that athletes living with their family tend to consume more probiotics as fermented foods such as yogurt and laban (a type of fermented drink) are often used in traditional meals. Conversely, meals provided by parents—whether for students on campus dining plans or those studying independently—may lack probiotic-rich options due to limited awareness.^(4)^

Prebiotic intake and living arrangements followed a similar trend, where athletes living in family homes also report higher consumption of fibre-rich foods — whole grains and vegetables. Those who are living alone or in dormitories may depend on processed meals with a decreased prebiotic content, which in turn reduces the gut microbiota diversity.^(34)^

### Implications

These findings highlight the need for targeted nutrition education tailored to athletes’ living arrangements, as dietary patterns and supplement use vary significantly between those living independently and those in family homes. Future research should explore how factors such as meal frequency, food accessibility, cooking skills, and the availability of probiotic- and prebiotic-rich foods influence gut health in student athletes. This is particularly relevant for athletes in dormitories or off-campus apartments, who may face greater challenges in maintaining gut-friendly dietary habits compared to those living in structured family environments. Educational programmes, particularly within university athletic departments, could be tailored to enhance knowledge and access to probiotic and prebiotic-rich foods.

### Strengths and limitations

This study provides valuable insight into the knowledge and dietary practices related to probiotics and prebiotics among a relatively large and diverse sample of Jordanian athletes, using a culturally adapted and psychometrically validated questionnaire. A key strength is its focus on an under-researched population in the Middle East, contributing to the global understanding of nutrition behaviours in athletic settings.

However, certain limitations should be acknowledged. The cross-sectional design precludes causal inference, and reliance on self-reported data may introduce recall or social desirability bias. Furthermore, the absence of objective clinical measurements — such as biomarkers of gut health or stool microbiota analysis — limits the ability to validate reported GI symptoms.

The lack of a control group or intervention component also restricts the ability to test the effectiveness of specific nutrition strategies. Finally, the sample was limited to athletes aged 18–22, which may affect the generalisability of the findings to older or professional athletic populations.

## Conclusion

This study aimed to assess the knowledge, dietary habits, and GI health of Jordanian athletes about probiotics and prebiotics. The findings revealed that Jordanian athletes had a limited understanding of probiotics and prebiotics. However, differences in certain knowledge areas and eating patterns were discovered. GI problems were common but not significantly linked with knowledge levels or diet categories. Symptoms and nutritional choices varied depending on the living situation.

These results underscore the need for tailored educational interventions in sports nutrition and emphasise the role of policymakers in collaborating with sports organisations to improve access to probiotic-rich foods and evidence-based dietary guidelines to support the health and performance of Jordanian athletes. Future studies should investigate the longer-term effects of these interventions and evaluate the effect of conditions such as training volume, hydration and stress on the occurrence of GI symptoms in this population.

## Data Availability

The data and materials supporting the findings of this study are available upon reasonable request.
